# Efficacy of Treatments in Nonarteritic Ischemic Optic Neuropathy: A Systematic Review and Meta-Analysis

**DOI:** 10.3390/ijerph19052718

**Published:** 2022-02-26

**Authors:** Krisztina Lantos, Zsuzsa Réka Dömötör, Nelli Farkas, Szabolcs Kiss, Zsolt Szakács, András Garami, Gábor Varga, László Lujber, Reem Kanaan, Péter Hegyi, Gergely Fehér, Valéria Gaál

**Affiliations:** 1 Department of Ophthalmology, Medical School, University of Pécs, 7632 Pécs, Hungary; lantos.krisztina@pte.hu; 2 Department of Ophthalmology, Medical School, University of Debrecen, 4032 Debrecen, Hungary; reka.zsuzsa@gmail.com; 3 Institute of Bioanalysis, Medical School, University of Pécs, 7624 Pécs, Hungary; farkas.nelli@gmail.com; 4 Doctoral School of Clinical Medicine, University of Szeged, 6720 Szeged, Hungary; kissszabolcs1995@gmail.com; 5 Institute for Translational Medicine, Medical School, University of Pécs, 7624 Pécs, Hungary; szaki92@gmail.com (Z.S.); andras.garami@aok.pte.hu (A.G.); hegyi.peter@pte.hu (P.H.); 6 Department of Oral Biology, Faculty of Dentistry, Semmelweis University, 1089 Budapest, Hungary; varga.gabor@dent.semmelweis-univ.hu; 7 Department of Otorhinolaryngology, Medical School, University of Pécs, 7621 Pécs, Hungary; lujber.laszlo@pte.hu; 8 Department of Public Health Medicine, Medical School, University of Pécs, 7624 Pécs, Hungary; rkanaan3@gmail.com; 9 Department of Primary Health Care, Medical School, University of Pécs, 7624 Pécs, Hungary; feher.gergely@pte.hu

**Keywords:** nonarteritic ischemic optic neuropathy, NAION, meta-analysis

## Abstract

Background: Nonarteritic Anterior Ischemic Optic Neuropathy (NAION) is the second most common cause of optic nerve-related permanent visual loss in adults. Aim: We aimed to analyze the efficacy of the noninvasive and minimally invasive therapeutic options of NAION. Methods: We performed a systematic literature search in MEDLINE, EMBASE, and CENTRAL from inception to 10 June 2019 to identify the studies that report on the effect of different therapies on visual acuity (VA) and visual field (VF). Weighted mean difference (WMD) with 95% confidence interval (CI) was calculated for these outcomes. The efficacy of steroids was investigated in quantitative, oxygen, steroid plus erythropoietin (EPO), levodopa/carbidopa, memantine, and heparin-induced extracorporeal LDL/fibrinogen precipitation (HELP) therapies and other therapeutic modalities in qualitative synthesis. Results: Thirty-two studies were found to be eligible. We found that steroid therapy compared to control did not improve VA (*p* = 0.182, WMD = 0.14, 95% CI: −0.07, 0.35) or VF (*p* = 0.853, WMD = 0.16, 95% CI: −1.54, 1.86). Qualitative analysis could be performed for oxygen, steroid plus EPO, and HELP as well, however, none of them showed VA and VF benefit. Two individual studies found memantine and levodopa beneficial regarding VA. Conclusion: Our systematic review did not reveal any effective treatment. Further investigations are needed to find therapy for NAION.

## 1. Introduction

Nonarteritic ischemic optic neuropathy (NAION) is the most common acute optic neuropathy in patients over 50 years of age. It affects 2 to 10 persons per 100,000 [1].

NAION is characterized by sudden painless loss of vision and visual field defects, an arcuate or altitudinal defect particularly in the inferior visual field or other patterns of nerve fiber bundle defect. Clinically, it is characterized by segmental swelling of the optic disc, which in several months leads to optic atrophy.

The exact etiology of NAION is unknown. The pathogenesis of NAION is generally accepted as a decrease in perfusion pressure to the optic nerve head resulting in acute ischemic infarction of the optic nerve and secondary inflammation, with later apoptosis of the retinal ganglion cells. Some authors believe that it shows an analogy with the etiopathogenesis of central nervous system multiple microembolism. Multiple embolisms occurring in the lateral end branch of the posterior ciliary artery, which supplies the optic disc, are most likely to play a central role in the development of the condition. Undetected or untreated hypertension, atheromatosis, and diabetes mellitus are the most important underlying diseases among patients suffering from NAION [2]. The fellow eye often becomes affected. Incidence of fellow eye NAION was 15% 5 years after the first eye was affected. In younger patients, the risk of fellow eye involvement seems to be higher with 35% involvement of the second eye within 7 months [3].

There is no generally accepted treatment for NAION. Most of the current medical therapeutic approaches are empirical, and mainly target the possible causes of ischemia, such as thrombosis, insufficient blood supply, and inflammation, or apply neuro-protective and neuro-regenerative agents, because retinal ganglion cell death is the final consequence.

The available literature of the possible treatment of NAION is quite diverse and controversial. Several trials evaluated the efficiency of steroids applying systemic (oral or intravenous) or intravitreal administration on NAION [1,4,5,6,7,8,9,10,11,12,13,14]. There are some studies of levodopa efficacy in the improvement of visual function in NAION patients [15,16,17,18]. Other studies addressed the neuroprotective actions of different agents, such as erythropoietin (EPO), brimonidine, and memantine, targeting the preservation of neuronal function [5,19,20,21,22].

The microcirculatory vasodilator PGE1 was also investigated as a therapeutic agent as well as anticoagulant drugs such as heparin and warfarin [7,23]. A significant improvement of the hemorheological abnormalities was described using heparin-induced extracorporeal LDL/fibrinogen precipitation (HELP), which eliminates fibrinogen, LDL, cholesterol, and triglycerides from blood, so the plasma viscosity decreases and the microcirculation improves. The effectiveness of HELP treatment was assessed by the visual function of patients affected by NAION [24,25,26]. Additionally, two published case series showed that intravitreal treatment with anti-VEGF agent (ranibizumab) [27,28] had beneficial effects for NAION. In contrast, another study did not find difference between intravitreal bevacizumab and the natural history in change of visual function in patients [29]. 

In the treatment of NAION, some studies have found results in surgical solutions such as optic nerve decompression, transvitreal optic neurotomy, and pars plana vitrectomy with removal of epipapillary adhesions. In the Ischemic Optic Neuropathy Decompression Trial (IONDT), the results of the treated eyes were worse than those of the untreated eyes and 42% of cases showed improvement in visual acuity spontaneously. Optic neurotomy and vitrectomy have been examined in a small number of studies and their results are not suitable for comparative analysis [3]. There is little chance of surgical solutions spreading, so the aim of this systematic review was to investigate the efficacy of noninvasive and minimally invasive treatment options for NAION.

## 2. Materials and Methods

Our meta-analysis was conducted according to the recommendations of the Cochrane Handbook for Systematic Reviews of Interventions and was registered in PROSPERO International Prospective Register of Systematic Reviews (registration number CRD42018102521). Preferred Reporting Items for Systematic Reviews and Meta-Analysis (PRISMA) guidelines were applied to report our results [30]. We deviated from the protocol in that we also narratively analyzed non-comparative studies, because we wanted to show a more complex view about therapeutic difficulties.

### 2.1. Eligibility Criteria

We created our scientific question following the population-intervention-control-outcomes (PICO) framework: (P) our population consisted of patients with nonarteritic anterior ischemic optic neuropathy, (I) who received a therapeutic intervention (corticosteroids or levodopa with carbidopa or erythropoietin, pentoxifylline, brimonidine, memantine, prostaglandin E1, ranibizumab, bevacizumab, oxygen, heparin-induced extracorporeal LDL/fibrinogen precipitation (HELP), Fasudil), (C) compared with no treatment or placebo, and our (O) outcomes were improvement of visual acuity (VA), change in visual field (VF), and retinal nerve fiber layer (RNFL) thickness. Studies were included in our qualitative synthesis if they reported the mentioned therapeutic interventions even if they were not comparative studies. Studies that used the Humphrey visual field analyzer were included in our quantitative analysis of VF. We compared the mean deviation (MD) values of these studies. Studies in which the treatment was not initiated within 1 month after the onset of NAION or that applied surgical interventions were excluded.

### 2.2. Search and Selection Strategy

Our systematic search was performed in MEDLINE (via PubMed), EMBASE and CENTRAL (Cochrane Central Register of Controlled Trials) from inception to 10 June 2019. Our search query was ‘((non-arteritic OR nonarteritic) AND anterior AND ischemic AND optic AND neuropathy) OR NA-AION OR N-AION OR NAION’. No search filters were applied.

The results of our search were imported to and processed with the EndNote X7.4 software (Clarivate Analytics, Philadelphia, PA, USA). After removing duplicates automatically and manually, the studies were screened by title, then by abstract, and finally by full text by two independent investigators (K.L., V.G.). Disagreements were resolved by consensus.

### 2.3. Data Extraction 

Numeric data were extracted independently by two reviewers (K.L. and V.G.) and entered into a purpose-designed Excel datasheet (Office 365, Microsoft, Redmond, WA, USA). We extracted data of the author of the study, year of publication, study design, details of the intervention, length of follow-up, number of patients, and the outcomes: VA, VF, and RNFL thickness, before the treatment and after at specified times. Any discrepancies were resolved by consensus.

### 2.4. Statistical Analysis 

For data synthesis, we used the methods recommended by the working group of the Cochrane Collaboration [31]. Random effects-models by DerSimonian and Laird [32] were used to conduct a meta-analysis to assess the effect of different therapies on VA and VF. In the case of VA as a continuous variable, weighted mean difference (WMD) and 95% confidence interval (CI) of logMAR values were estimated. The VA was reported in LogMAR values in all but one study, in which data had to be converted to LogMAR values [33].

For VA as a categorical variable, ‘improved’ and ‘not improved’ categories were used to calculate pooled odds ratios with 95% CI. In case of VF as a continuous variable WMD and 95% CI of mean deviation values were estimated. Because in some studies there were no events observed, we performed a continuity correction recommended in the Cochrane Handbook and proposed by Sweetin et al. [34] to overcome the difficulty of dividing by 0. We calculated WMD for the therapies and outcomes with sufficient data for the analysis. The other studies were summarized narratively.

When the number of studies was sufficient for statistical analysis, publication bias was evaluated by visual inspection of funnel plots and test f H0. Heterogeneity was tested using Cochrane’s Q and I2 statistics.

We performed all meta-analytic calculations with STATA 16 statistical software (STATA Corp. 2019. Stata Statistical Software: Release 16. College Station, TX, USA: StataCorp LLC.).

### 2.5. Risk of Bias Assessment

The risk of bias assessment was done at the study level and then summarized narratively. We used the revised Cochrane Collaboration’s risk-of-bias tools for randomized trials—RoB 2 tool [35] and non-randomized trials—ROBINS-I tool [36]. The tools were assessed by three investigators (K.L., V.G., Z.R.D.). Disagreements were resolved by consensus.

### 2.6. Quality of Evidence

The Grades of Recommendation Assessment, Development, and Evaluation (GRADE) approach was used to assess the certainty of evidence regarding the outcomes. [37]. It was assessed by two investigators (K.L., Z.R.D.).

## 3. Results

### 3.1. Study Selection and Characteristics of the Studies Included

**Figure 1:**
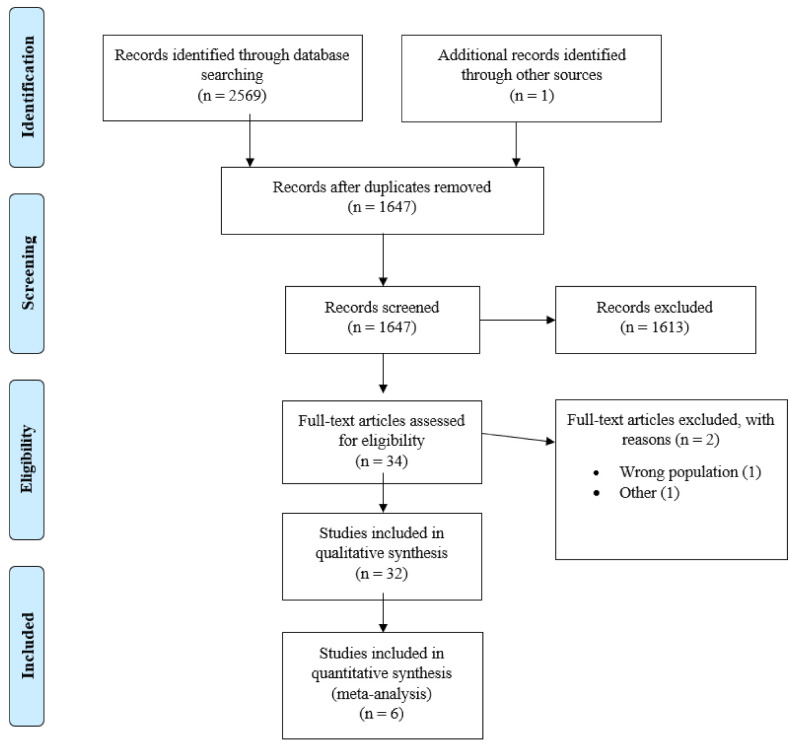
PRISMA Flowchart.

The results of the literature search are illustrated by the flowchart in Figure 1. A total of 2570 articles were identified and 32 of these were included in qualitative synthesis and 6 of these with 524 patients in quantitative analysis. The summary of the characteristics of the studies included in our analysis is shown in Table 1.

Most of the included studies contained data on the change in the VA during the study period. Fewer studies provided data on the change between the initial and final VA values. Due to the lack of RNFL thickness data in most of the included studies, we could not analyze this outcome.

### 3.2. Effects of Steroid Therapy on Visual Acuity

**Figure 2:**
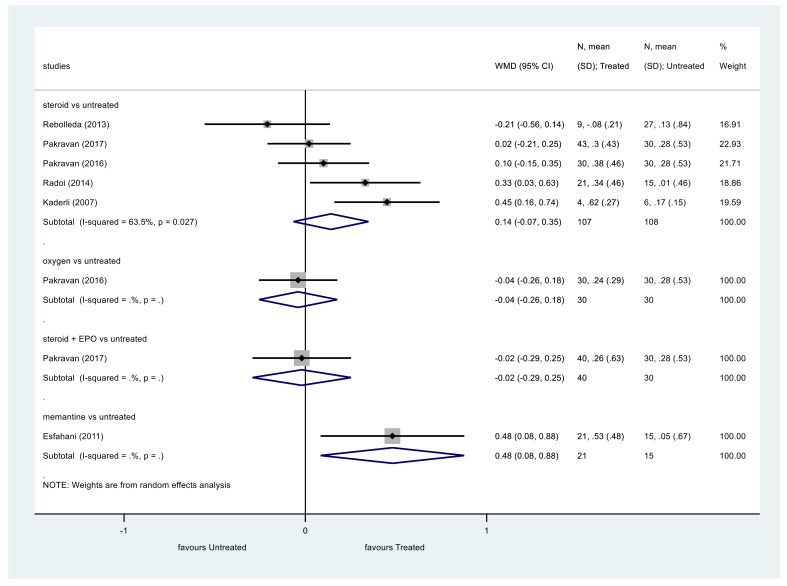
Comparison of interventions to no treatment regarding visual acuity (as a continuous variable).

First, we analyzed VA as a continuous variable (Figure 2). We imported or converted every VA value in LogMAR and in all the studies included, the follow-up period lasted for at least 6 months.

Data regarding steroid therapy showed that steroids did not significantly improve VA compared to the control group (*p* = 0.182, WMD = 0.14, 95% CI: −0.07, 0.35). Heterogeneity among these studies was moderate (I^2^ = 63.5%, *p* = 0.027).

**Figure 3:**
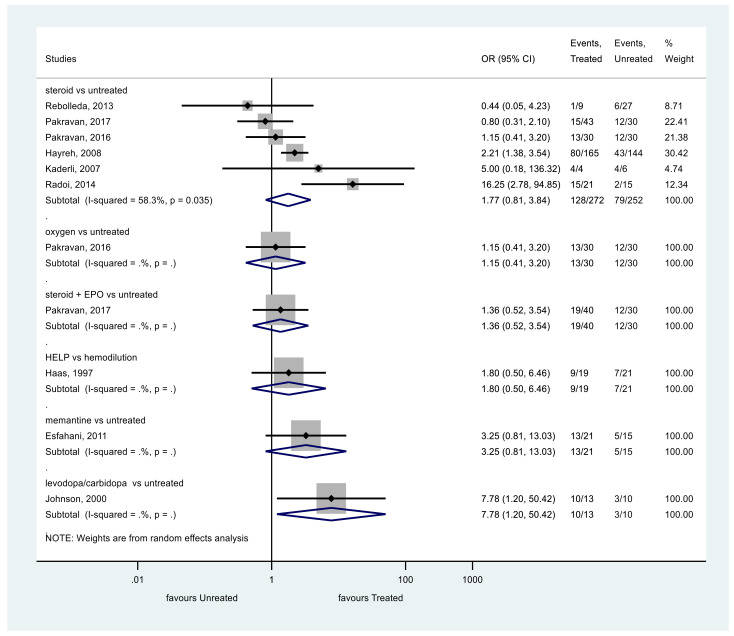
Comparison of interventions to no treatment regarding visual acuity (as a categorical variable).

The same results were obtained analyzing VA as a categorical variable (Figure 3). The VA of patients treated with steroids did not show significant improvement at the end of the follow-up compared to the control group (*p* = 0.149, OR = 1.77, 95% CI: 0.81, 3.84). Heterogeneity was moderate among these studies, too (I^2^ = 58.3%, *p* = 0.035).

### 3.3. Effects of other Therapies on Visual Acuity

We identified only one eligible study investigating the effects of oxygen therapy on VA. In this study, oxygen therapy did not significantly improve the VA of patients compared to the control group [1].

We included one article which investigated the effect of combined intravenous erythropoietin (EPO) and corticosteroid therapy. Steroid plus EPO did not improve the VA significantly [5].

One eligible study investigated the effect of memantine therapy [22]. Analyzing the results as continuous variables memantine showed improved VA compared to the control group, but as a categorical variable, memantine did not significantly improve the VA [22].

We identified a study that compared the effect of levodopa with carbidopa to placebo on VA. Johnson et al. revealed a significant improvement of VA in the levodopa, carbidopa group [17].

Lyttle et al. found that levodopa with carbidopa improved the central VA of the participants with an initial VA of 20/60 or worse. The mean change in VA of the patients with better VA was not reported so we could not include these data in our analysis. Due to inconsistent data reporting in tables vs. plain text, we did not include their results in our analysis of VA as a categorical variable [18].

Haas et al. investigated the effect of heparin-induced extracorporeal LDL/fibrinogen precipitation. The improvement of VA did not differ significantly between the HELP and the hemodilution group [24].

During categorization of the eligible studies, two articles [10,17] could not be used, because patients with better than 20/40 initial VA had no further data on the improvement of VA. We conducted a meta-analysis without the study by Hayreh et al. [10,38] and the result was very similar without a significant decrease in the heterogeneity among the studies (Supplementary Figure S1).

### 3.4. Effects of Therapies on Visual Field (VF)

**Figure 4:**
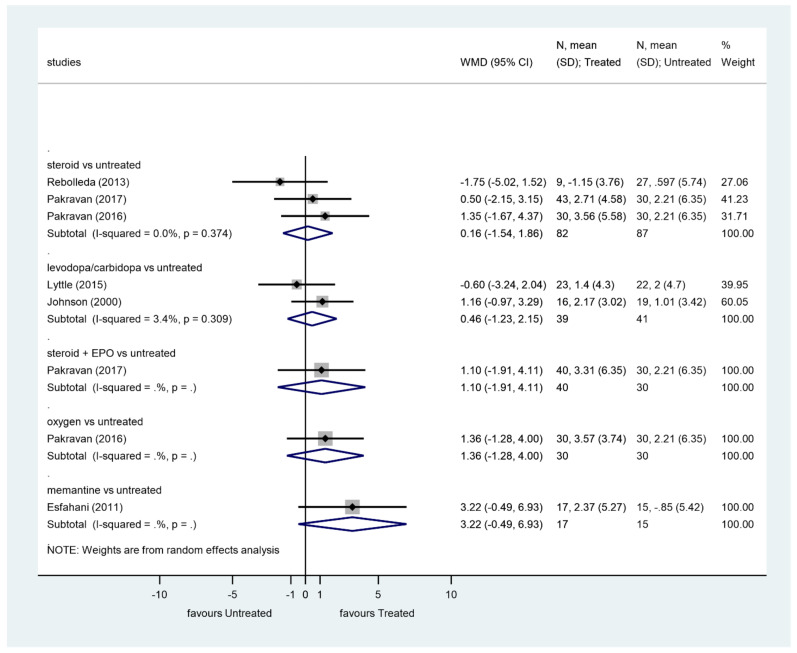
Comparison of interventions to no treatment regarding visual field.

We analyzed the effect of treatments on VF in studies having at least 3 months of follow-up (Figure 4).

Steroids did not significantly improve VF compared to the control group (*p* = 0.853, WMD = 0.16, 95% CI: −1.54, 1.86). There was no heterogeneity among these studies (I^2^ = 0.0%, *p* = 0.374).

Levodopa/carbidopa did not significantly improve VF compared to the control group (*p* = 0.596, WMD = 0.46, 95% CI: −1.23, 2.15). Heterogeneity among these studies was very low (I^2^ = 3.4%, *p* = 0.309).

There was no significant improvement of VF after steroid plus EPO therapy, oxygen therapy, or memantine therapy.

Our results are represented in a summary table (Table 2).

### 3.5. GRADE of Evidence, Risk of Bias Assessment, and Publication Bias

The results of the evidence grading are shown in Table 3, Table 4 and Table 5. The results of the risk of bias assessment are shown in Supplementary Tables S1 and S2.

In case of VA as a continuous variable, due to low study numbers, publication bias was assessed only in the group of studies with steroid therapy. The result of the visual assessment and test of H0 (*p* = 0.926) revealed no small study effect (Supplementary Figure S2). Similarly, the result of the visual assessment of the funnel plot and test of H0 (*p* = 0.983) revealed no small study effect in case of VA as a categorical variable (Supplementary Figure S3). We could not assess the publication bias in case of VF, due to the low number of studies.

## 4. Discussion

In our systematic review and meta-analysis, we summarized the findings that are currently available in clinical literature of the effects of therapeutic modalities on the outcomes of NAION.

### 4.1. Steroid and NAION

Corticosteroids have antiedematous, antiphlogistic effects, can decrease capillary permeability, and decrease compression of capillaries in the optic nerve head, improving blood flow and restore the function of surviving ischemic axons in NAION. [43] Our meta-analysis of 6 studies for VA and 3 for VF demonstrated that steroids did not improve VA and VF significantly. However, the results of a study by Hayreh et al. [10] provided support for the beneficial effect of steroids. They found that oral corticosteroid therapy resulted in a significantly higher probability of improvement in VA. Two studies with intravitreal steroid therapy (triamcinolone injection) [8,9] showed significant improvement of VA and VF, although one of them had a small number of cases [9]. The effect of steroid and pentoxifylline was also described in a study showing that fluocortolone in combination with pentoxifylline has a beneficial effect on VA, but there was no significant difference in the VF [14]. In contrast to the aforementioned studies, Rebolleda et al. and Kinori et al. reported no functional difference between the steroid and the untreated groups [4,6]. Moreover, a randomized, double-blind clinical trial supports our findings, as it concluded that steroids did not improve the VA significantly at 6 months. Unfortunately, we could not include this study in our meta-analysis due to missing data about the initial and final VA and VF values of the patient groups [11]. Pakravan et al. evaluated the efficacy of normobaric oxygen therapy in addition to steroids [1]. Their findings did not reveal beneficial effects of either steroids or oxygen for the management of NAION compared to placebo. Steigerwalt et al. used PGE1 with steroids [7], but we did not include it in our analysis because the control group also received steroids. They found that VA improved in the cases treated with PGE1 compared to the control group. We found a meta-analysis published by Chen et al. which investigated only steroid therapy in NAION. Their article also supports the results of our meta-analysis, that steroids do not significantly improve VA [43]. Our meta-analysis investigated not only steroid therapy but we also examined the VF in addition to VA. Our results suggest that steroids did not significantly improve VA or VF in NAION.

### 4.2. Levodopa/Carbidopa and NAION

Levodopa crosses the blood–retinal barrier to increase retinal dopamine level. Dopamine is a neurotransmitter, neuromodulator, and neuroprotective agent. There are some studies about the effects of levodopa on visual function in patients with NAION. Lyttle et al. found that levodopa improved central VA [18]. Johnson et al. [17] published VA improvement results in patients with 20/40 VA or worse, 76.9% in the levodopa group and 30% of the control group had improved VA. Johnson et al. [15] found improvement of VA among patients receiving levodopa and carbidopa despite a long-standing visual loss; however, this study refers to earlier publications, which stated that visual improvement might have been occurred because of the spontaneous resolution of NAION. In contrast with what Johnson found, in the study by Simsek et al., there was no improvement in VA either in the study or the placebo group, suggesting that levodopa and carbidopa therapy cannot restore a long-standing visual loss [16]. Unfortunately, these studies could not meet our eligibility criteria for the quantitative synthesis, therefore we could not perform the meta-analysis of their results.

### 4.3. EPO and NAION

Moderres et al. published a study where 31 patients received intravitreal injection of erythropoietin solution and it showed improvement in VA. Neuroprotection is a therapeutic strategy in the treatment of NAION. EPO reduces apoptosis in retinal ganglion cells [19]. Pakravan et al. [5] compared the effect of steroid therapy alone or in combination with systemic EPO for the treatment of NAION. They found no beneficial effect in either group, similar to our results.

### 4.4. Brimonidine and NAION

Topical brimonidine tartrate is an alpha-adrenergic agonist agent, which has a neuroprotective effect for retinal ganglion cells. We found two studies [20,21] that examined the effects of brimonidine tartrate as a treatment of NAION, but they did not find an improvement of visual function. Wilhelm’s double-masked, randomized, placebo-controlled trial was not included in our analysis due to the ambiguity in the patient number in the treatment groups.

### 4.5. Memantine and NAION

Memantine is a noncompetitive NMDA receptor antagonist and it relieves glutamate NMDA-receptor mediated toxicity in retinal ganglion cells. Analyzing the results of Esfahani et al. [22] as a continuous variable we found that memantine improves VA compared to the control group.

### 4.6. Heparin-Induced Extracorporeal LDL/Fibrinogen Precipitation and NAION

HELP improves rheologic status of tissues. We found four publications about HELP and hemodilution [24,25,26,40]; one of these was analyzed statistically, a prospective, randomized, controlled study by Haas et al., which suggested the HELP system is more effective than hemodilution in the treatment of NAION.

### 4.7. Anticoagulants and Thrombolytics

Multiple embolization may play a role in the development of NAION. We found publications investigating the efficacy of anticoagulants and thrombolytics. The recanalization rate in response to thrombolytic therapy improves as a vessel narrows [44]. Aftab et al. found that patients with NAION did benefit from anticoagulation with heparin and warfarin [23].

### 4.8. Limitations

We found few studies that we could use in quantitative analysis. There are no large uniform multicenter studies to compare. We included only two RCTs in addition to other comparative studies in our quantitative analysis of steroid therapy. Most of them had a small sample size and there were differences in the route, administration intervals and dosage of interventions. These differences probably explain the moderate data heterogeneity among these studies. As mentioned before, two studies [10,17] did not report the change in VA of their patients with VA better than 20/40, therefore we were unable to include these data in our meta-analysis. This could explain the heterogeneity in our results in that group.

## 5. Conclusions

There are many medical therapies in practice, but based on our meta-analysis and systematic review, we did not find the drug that is generally recommended. Further randomized, controlled trials are necessary to find the most effective therapy for NAION. NAION has a number of etiological factors and there may be no general therapeutic option. Subgroups of different etiologies should be considered separately.

With eliminating general cardiovascular predisposing factors, we can reduce the incidence of NAION.

Further human investigation and animal experiences are needed to help us to understand the earliest pathological processes and to find the effective therapy for NAIONs.

## Figures and Tables

**Table 1:** Characteristics of the studies included.

Study, Year	Study Design	Interventions	No.of Participants	VA Follow-Up (Months)
Rebodella et al., 2013 [4]	retrospective cohort study	prednisolone PO	10	6
		untreated	27	
Pakravan et al., 2016 [1]	randomized clinical trial	iv. methylprednisolone, prednisolone PO	30	6
		100% normobaric oxygen	30	
		untreated	30	
Kinori et al., 2014 [6]	retrospective cohort study	iv. methylprednisolone, prednisolone PO	24	22
		untreated	24	36
Steigerwalt et al., 2008 [7]	prospective cohort study	i.v methylprednisolone+ PGE1	8	6
		prednisolone PO	7	
Pakravan et al., 2017 [5]	prospective cohort study	iv. methylprednisolone + EPO, prednisolone PO	40	6
		iv. methylprednisolone, prednisolone PO	43	
		untreated	30	
Radio et al., 2014 [8]	retrospective cohort study	intravitreal triamcinolone	21	6
		untreated	15	
Kaderli et al., 2007 [9]	retrospective cohort study	intravitreal triamcinolone	4	12–15
		untreated	6	9–12
Hayreh et al., 2008/1 [10]	retrospective cohort study	prednisolone PO	312	6
Hayreh et al., 2008/2 [38]		untreated	301	
Saxena et al., 2018 [11]	randomized, double-blind placebo-controlled trial	prednisolone PO	19	6
		untreated	19	
Prokosch et al., 2014 [14]	randomized controlled trial	iv+per os pentoxifylline	30	6
		iv+per os pentoxifylline + fluocortolone	30	
Vidovic et al., 2015 [12]	prospective case series	methylprednisolone PO	38	6
Yaman et al., 2008 [13]	case series	intravitreal triamcinolone	4	3
Modarres et al., 2011 [19]	prospective case series	intravitreal EPO	31	6
Johnson et al., 1996 [15]	randomized, double-masked placebo-controlled trial	levodopa/carbidopa	10	6
		untreated	10	
Lyttle et al., 2015 [18]	retrospective cohort study	levodopa/carbidopa	33	8
		untreated	26	
Simsek et al., 2005 [16]	randomized, placebo-controlled trial	levodopa/carbidopa	12	11
		untreated	12	10
Johnson et al., 2000 [17]	retrospective cohort study	levodopa/carbidopa	18	6
		untreated	19	
Bajin et al., 2011 [27]	retrospective case series	intravitreal ranibizumab	4	3
Saatsi et al., 2013 [28]	retrospective case series	intravitreal ranibizumab	17	12
Prescott et al., 2012 [39]	retrospective case series	intravitreal bevacizumab	5	inconsistent
Rootman et al., 2013 [29]	non-randomized controlled trial	intravitreal bevacizumab	17	6
		untreated	8	
Fazzone et al., 2003 [20]	retrospective cohort study	topical brimonidine	14	2–3
		untreated	17	
Wilhelm et al., 2006 [21]	randomized, double masked, placebo-controlled trial	topical brimonidine	11	3–3,5
		untreated	18	
Haas et al., 1997 [24]	randomized, controlled trial	HELP	19	3
		hemodilution	21	
Ramunni et al., 2005 [25]	case series	HELP	11	3
Haas et al., 1994 [40]	retrospective case series	hemodilution	24	24
Guerriero et al., 2009 [26]	prospective case series	LDL apheresis	10	6
		conventional therapy	10	
Bojic et al., 1994 [41]	case series	hyperbaric oxygen	9	6
Aftab et al., 2006 [23]	prospective interventional pilot study	iv Heparin, Warfarin PO	24	6
Sanjari et al., 2016 [42]	case series	intravitreal Fasudil	13	3
Esfahani et al., 2011 [22]	randomized, double-masked controlled trial	memantine PO	25	6
		untreated	22	

**Table 2:** Summary table of results.

Therapy	VA (as Continuous Variable)	VA (as Categorical Variable)	VF
Steroid	Did not improve	Did not improve	Did not improve
oxygen	Did not improve	Did not improve	Did not improve
EPO+steroid	Did not improve	Did not improve	Did not improve
memantine	Improved	Did not improve	Did not improve
Levodopa/carbidopa		Improved	Did not improve
HELP		Did not improve	

**Table 3:** GRADE of evidence of our results for visual acuity as a continuous variable.

Outcomes	Anticipated Absolute Effects * (95% CI)		Relative Effect (95% CI)	No. of Participants (Studies)	Certainty of the Evidence (GRADE)	Comments
	Risk with Untreated	Risk with Treated				
Steroid vs. untreated follow up: range 6 months to 15 months	The mean steroid vs. untreated was 0 logMAR	WMD 0.14 logMAR higher (0.07 lower to 0.35 higher)	**-**	215 (5 observational studies)	⨁◯◯◯ VERY LOW ^a^^,^^b^^,^^c^	
Oxygen vs. untreated	The mean oxygen vs. untreated was 0	WMD 0.04 lower (0.26 lower to 0.18 higher)	-	60 (1 RCT)	⨁◯◯◯ VERY LOW ^d^^,^^e^	
Steroid+EPO vs. untreated	The mean steroid+EPO vs. untreated was 0	WMD 0.02 lower (0.29 lower to 0.25 higher)	-	70 (1 observational study)	⨁◯◯◯ VERY LOW ^d^^,^^e^	
Memantine vs. untreated	The mean memantine vs. untreated was 0	WMD 0.48 higher (0.08 higher to 0.88 higher)	-	36 (1 RCT)	⨁◯◯◯ VERY LOW ^d^^,^^e^	
**GRADE Working Group grades of evidence** **High certainty**: We are very confident that the true effect lies close to that of the estimate of the effect. **Moderate certainty:** We are moderately confident in the effect estimate: The true effect is likely to be close to the estimate of the effect, but there is a possibility that it is substantially different. **Low certainty:** Our confidence in the effect estimate is limited: The true effect may be substantially different from the estimate of the effect. **Very low certainty:** We have very little confidence in the effect estimate: The true effect is likely to be substantially different from the estimate of effect.						

Explanations: a. Among these 5 studies, there were 2 with low risk of bias, 2 with moderate, and 1 with serious risk of bias. b. Heterogeneity among the studies was substantial (I2 = 63.5%, *p* = 0.027). c. Interventions delivered differently in different settings. d. Not applicable. e. Low sample size. * The risk in the intervention group (and its 95% confidence interval) is based on the assumed risk in the comparison group and the relative effect of the intervention (and its 95% CI). CI: Confidence interval.

**Table 4:** GRADE of evidence of our results for visual acuity as a categorical variable.

Outcome No. of Participants (Studies)	Relative Effect (95% CI) *	Anticipated Absolute Effects (95% CI)			Certainty
		Control	Treated	Difference	
Steroid vs. untreated—assessed as a categorical variable (Visual Acuity) follow up: range 6 months to 15 months № of participants: 544 (6 observational studies)	OR 1.77 (0.81 to 3.84)	29.0%	42.0% (24.9 to 61.1)	13.0% more (4.1 fewer to 32.1 more)	⨁◯◯◯ VERY LOW ^a,b,c^
Oxygen vs. untreated № of participants: 60 (1 RCT)	OR 1.15 (0.41 to 3.20)	40.0%	43.4% (21.5 to 68.1)	3.4% more (18.5 fewer to 28.1 more)	⨁◯◯◯ VERY LOW ^d,e^
Steroid+EPO vs. untreated № of participants: 70 (1 observational study)	OR 1.36 (0.52 to 3.54)	40.0%	47.6% (25.7 to 70.2)	7.6% more (14.3 fewer to 30.2 more)	⨁◯◯◯ VERY LOW ^d,e^
HELP vs. hemodilution № of participants: 40 (1 observational study)	OR 1.80 (0.50 to 6.46)	33.3%	47.4% (20 to 76.4)	14.0% more (13.3 fewer to 43 more)	⨁◯◯◯ VERY LOW ^d,e^
Memantine vs. untreated № of participants: 36 (1 RCT)	OR 3.25 (0.81 to 13.30)	33.3%	61.9% (28.8 to 86.9)	28.6% more (4.5 fewer to 53.6 more)	⨁◯◯◯ VERY LOW ^d,e^
Levodopa/carbidopa vs. untreated № of participants: 23 (1 observational study)	OR 7.78 (1.20 to 50.42)	30.0%	76.9% (34 to 95.6)	46.9% more (4 more to 65.6 more)	⨁◯◯◯ VERY LOW ^d,e^
**GRADE Working Group grades of evidence** **High certainty:** We are very confident that the true effect lies close to that of the estimate of the effect. **Moderate certainty:** We are moderately confident in the effect estimate: The true effect is likely to be close to the estimate of the effect, but there is a possibility that it is substantially different. **Low certainty:** Our confidence in the effect estimate is limited: The true effect may be substantially different from the estimate of the effect. **Very low certainty:** We have very little confidence in the effect estimate: The true effect is likely to be substantially different from the estimate of effect.					

Explanations: a. Among these 6 studies, there were 3 with low risk of bias, 2 with moderate, and 1 with serious risk of bias. b. Heterogeneity among the studies was moderate (I2 = 58.3%, *p* = 0.035) probably due to the differences in the intervention and study designs. c. Interventions delivered differently in different settings. d. Not applicable. e. Low sample size. * The risk in the intervention group (and its 95% confidence interval) is based on the assumed risk in the comparison group and the relative effect of the intervention (and its 95% CI). CI: Confidence interval; OR: Odds ratio.

**Table 5:** GRADE of evidence of our results for visual field.

Outcomes	Anticipated Absolute Effects * (95% CI)		Relative Effect (95% CI)	No. of Participants (Studies)	Certainty of the Evidence (GRADE)	Comments
	Risk with Untreated	Risk with Treatment				
Pentoxiphilline+steroid vs. pentoxyphilline	The mean pentoxiphilline+steroid vs. pentoxyphilline was 0	WMD 3.2 lower (4 lower to 2.4 lower)	-	49 (1 observational study)	⨁◯◯◯ VERY LOW ^a,b,c^	
Steroid vs. untreated	The mean steroid vs. untreated was 0	WMD 0.16 higher (1.54 lower to 1.86 higher)	-	169 (3 observational studies)	⨁⨁◯◯ LOW ^c,d^	
Levodopa/carbidopa vs. untreated	The mean levodopa/carbidopa vs. untreated was 0	WMD 0.46 higher (1.23 lower to 2.15 higher)	-	80 (2 observational studies)	⨁◯◯◯ VERY LOW ^c,e^	
Steroid+EPO vs. untreated	The mean steroid+EPO vs. untreated was 0	WMD 1.1 higher (1.91 lower to 4.11 higher)	-	70 (1 observational study)	⨁◯◯◯ VERY LOW ^b,c^	
Oxygen vs. untreated	The mean oxygen vs. untreated was 0	WMD 1.36 higher (1.28 lower to 4 higher)	-	60 (1 RCT)	⨁◯◯◯ VERY LOW ^b,c^	
Memantine vs. untreated	The mean memantine vs. untreated was 0	WMD 3.22 higher (0.49 lower to 6.93 higher)	-	32 (1 RCT)	⨁◯◯◯ VERY LOW ^b,c^	
**GRADE Working Group grades of evidence** **High certainty:** We are very confident that the true effect lies close to that of the estimate of the effect. **Moderate certainty:** We are moderately confident in the effect estimate: The true effect is likely to be close to the estimate of the effect, but there is a possibility that it is substantially different. **Low certainty:** Our confidence in the effect estimate is limited: The true effect may be substantially different from the estimate of the effect. **Very low certainty:** We have very little confidence in the effect estimate: The true effect is likely to be substantially different from the estimate of effect.						

Explanations: a. Moderate risk of bias. b. Not applicable. c. Low sample size. d. There were minor differences in the dosage of the steroid therapy. e. As the results of our risk of bias assessment, both of the studies are at moderate risk of bias. * The risk in the intervention group (and its 95% confidence interval) is based on the assumed risk in the comparison group and the relative effect of the intervention (and its 95% CI). CI: Confidence interval.

## Supplementary Materials

The following supporting information can be downloaded at: https://www.mdpi.com/article/10.3390/ijerph19052718/s1, Table S1. Results of risk of bias assessement of randomized trials with RoB 2; Table S2. Results of risk of bias assessment in non-randomized trials with ROBIN-I tool; Figure S1. Forest plot of visual acuity, without Hayreh et al. (as a categorical variable); Figure S2. Funnel plot of visual acuity, as a continuous variable; Figure S3. Funnel plot of visual acuity, as a categorical variable
