# Supplementary material for: Efficacy of Treatments in Nonarteritic Ischemic Optic Neuropathy: A Systematic Review and Meta-Analysis
Source: Int J Environ Res Public Health. 2022 Feb 26;19(5):2718. doi: 10.3390/ijerph19052718 (PMC8910678; doi:10.3390/ijerph19052718)
Supplement: Supplementary file 1 [file ijerph-19-02718-s001.zip › ijerph-1525189-supplementary.pdf]

Supplementary Table S1. Results of risk of bias assessement of randomized trials with RoB 2.

**Studies with intention-to-treat**<br>**Author, Year**	**Experimental**	**Comparator**	Randomization process	Deviations from intended interventions	Missing outcome data	Measurement of the outcome	Selection of the reported result	Overall
Saxena, 2018	oral steroid	placebo	+	+	+	+	?	+
Wilhelm, 2006	brimonidine	placebo	+	+	+	+	?	+
Pakravan, 2016	steroid	oxygen and placebo	+	+	+	+	+	+
Esfahani, 2011	memantine	placebo	+	+	+	+	+	+
Johnson, 1996	levodopa, carbidopa	placebo	+	+	+	+	?	+
Haas, 1997	HELP	hemodilution therapy	+	+	+	+	?	+
Simsek, 2005	levodopa, carbidopa	placebo	?	?	+	+	?	!

+ Low risk

? Some concerns

– High risk

Supplementary Table S2. Results of risk of bias assessment in non-randomized trials with ROBIN-I tool.

Study		Pre-intervention		At-intervention	Post-intervention				Overall risk of bias
First author	Year	Bias due to confounding	Selection of participants	Classification of interventions	Deviation from intended interventions	Missing data	Mesurement of Outcomes	Selection of the reported results	
Rebolleda	2012	low	moderate	serious	Low	low	low	no information	moderate
Kinori	2014	low	moderate	moderate	low	low	low	no information	low
Steigerwalt	2008	moderate	moderate	serious	low	low	low	no information	moderate
Vidovic	2015	low	low	low	low	low	low	no information	low
Prokosch	2014	low	moderate	low	low	moderate	low	no information	moderate
Pakravan	2017	low	moderate	low	low	low	low	no information	low
Modarres	2011	low	moderate	moderate	low	low	low	no information	moderate
Radoi	2014	low	moderate	moderate	low	low	low	no information	moderate
Kaderli	2007	moderate	moderate	moderate	low	low	serious	no information	moderate
Rootman	2013	moderate	low	moderate	low	moderate	low	low	moderate
Fazzone	2003	low	serious	serious	low	low	low	no information	serious
Lyttle	2016	moderate	moderate	moderate	low	modetate	low	no information	moderate
Johnson	2000	low	moderate	moderate	low	moderate	low	no information	moderate
Guerriero	2009	no information	low	no information	no information	low	low	no information	moderate
Ramunni	2004	moderate	moderate	moderate	low	low	low	no information	moderate
Bajin	2011	low	moderate	low	low	low	low	no information	low
Saatci	2013	low	moderate	moderate	low	low	low	no information	low
Prescott	2012	moderate	serious	serious	low	low	low	no information	serious
Haas	1994	low	serious	moderate	low	low	low	no information	moderate
Bojic	1993	moderate	serious	serious	low	low	low	no information	serious
Aftab	2016	low	low	moderate	low	low	low	no information	low
Yaman	2008	low	no information	low	low	low	low	no information	low
Sanjari	2016	low	moderate	low	low	low	low	no information	low
Hayreh	2008	low	moderate	low	low	low	low	no information	low
Hayreh	2008	low	moderate	not applicable	not applicable	low	low	no information	low

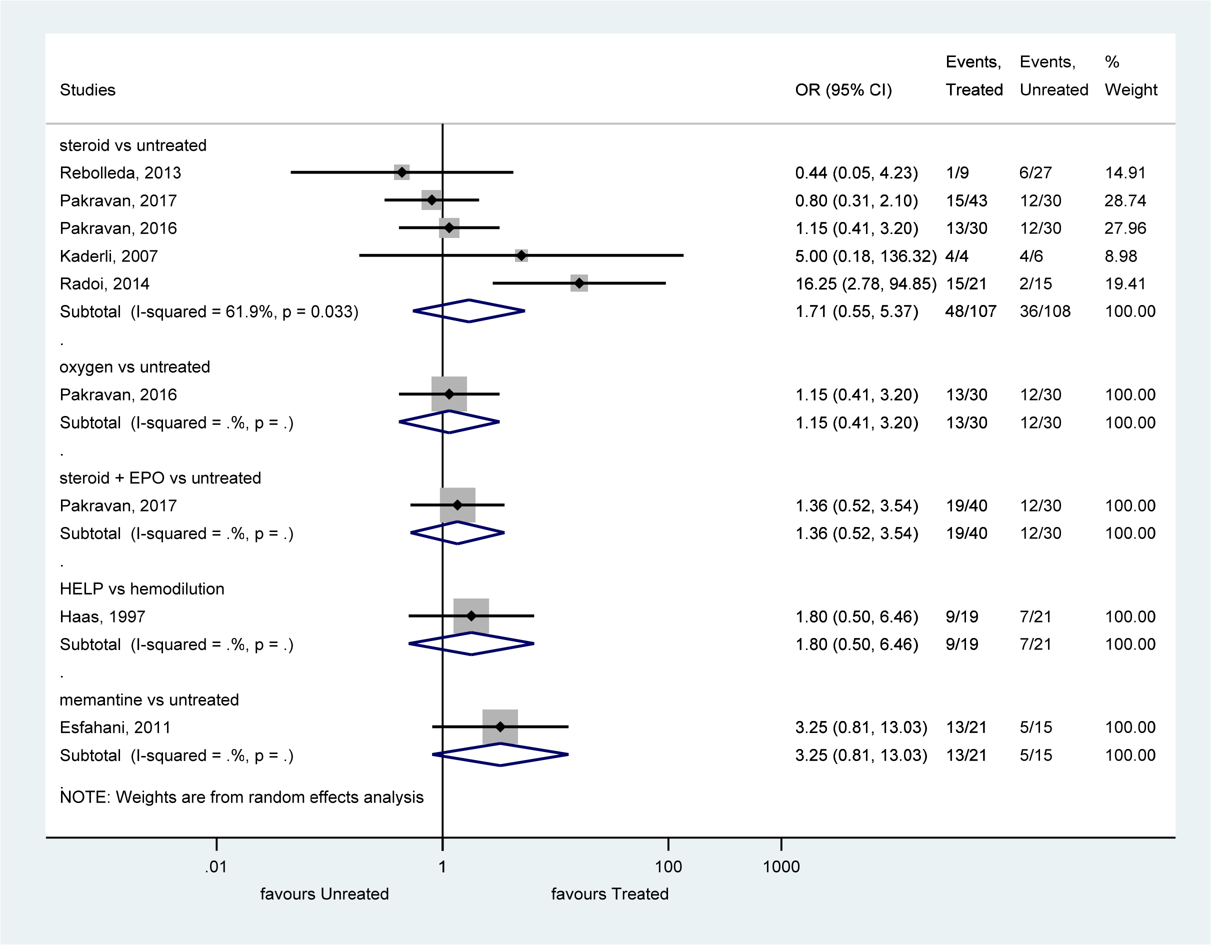

Supplementary Figure S1. Forest plot of visual acuity, without Hayreh et al. (as a categorical variable).

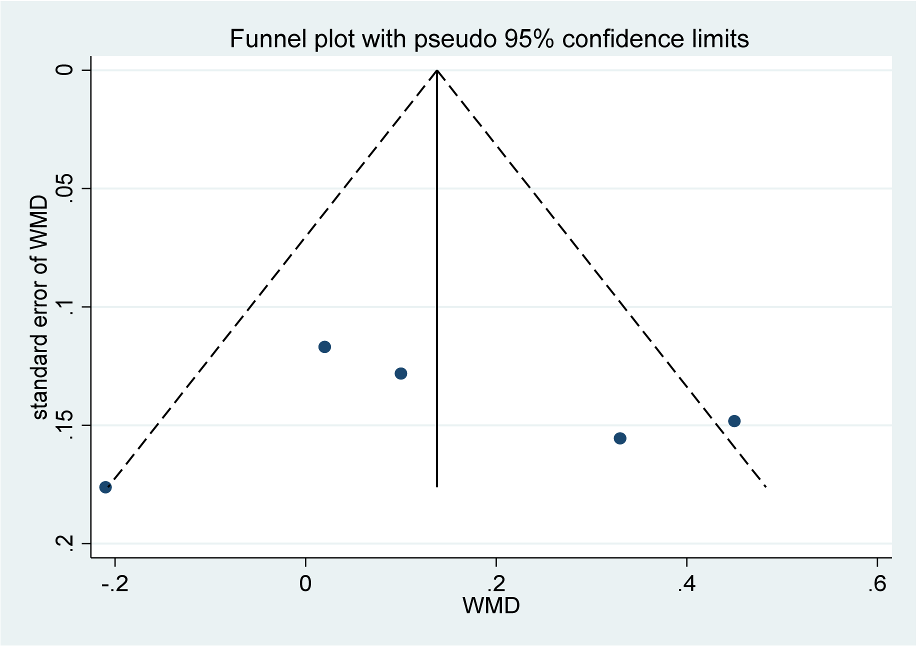

Supplementary Figure S2. Funnel plot of visual acuity, as a continuous variable.

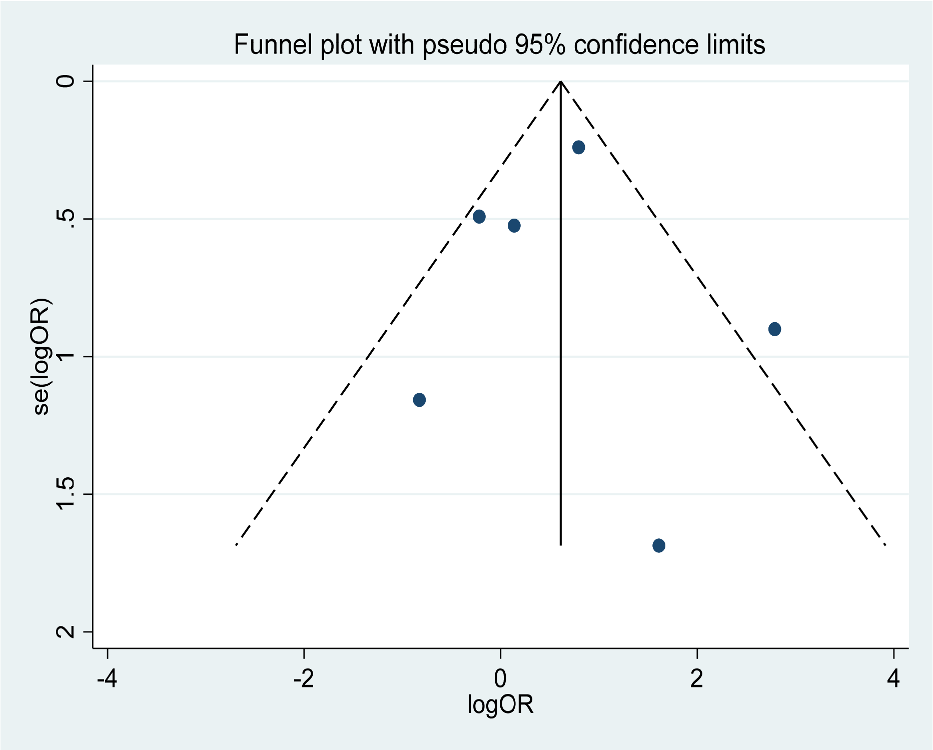

Supplementary Figure S3. Funnel plot of visual acuity, as a categorical variable.
